# Supplementary figures and images for: Association between physical activity duration and depressive symptoms in adolescents: A longitudinal study in a rural city in Japan
Source: PLoS One. 2024 May 31;19(5):e0304783. doi: 10.1371/journal.pone.0304783 (PMC11142661; doi:10.1371/journal.pone.0304783)

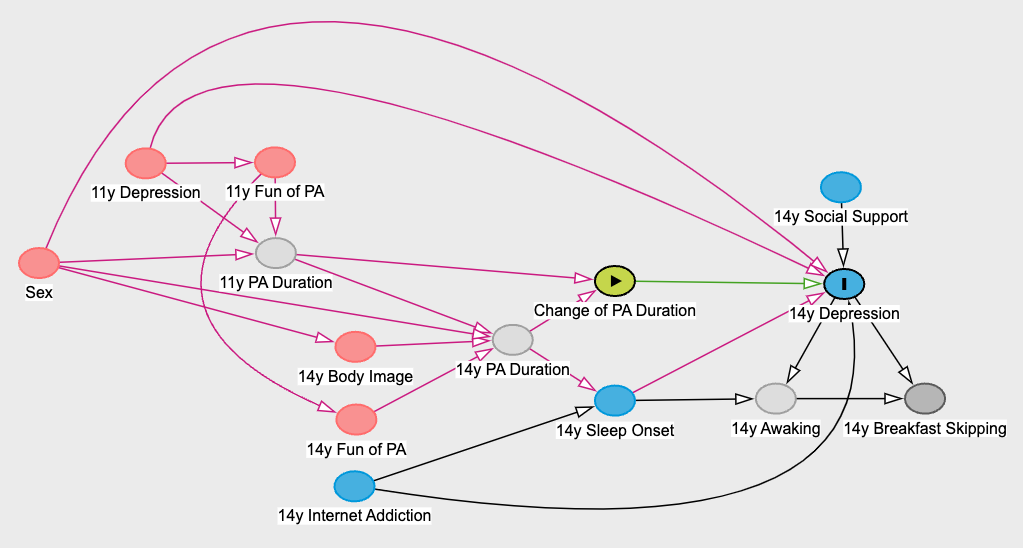

Supplement: S1 Fig — We made the DAG using the online resource "DAGitty" (https://www.dagitty.net/). We derived the variables and their relationships included in this DAG from previous studies and discussions with the co-author. (TIF) [file pone.0304783.s001.tif]
