# Supplementary material for: Association between physical activity duration and depressive symptoms in adolescents: A longitudinal study in a rural city in Japan
Source: PLoS One. 2024 May 31;19(5):e0304783. doi: 10.1371/journal.pone.0304783 (PMC11142661; doi:10.1371/journal.pone.0304783)
Supplement: Attachment — Submitted filename: Response_to_Reviewers.docx [file pone.0304783.s002.docx]

March 10, 2024

Dr. Mukhtiar Baig

Editor

*PLoS One*

Dear Dr. Baig,

Re: Resubmission of manuscript No. PONE-D-23-34588

We wish to re-submit the manuscript titled "Association between physical activity time and depressive symptoms in adolescents: A longitudinal study in a rural city in Japan."

We sincerely thank you and the reviewers for their careful assessment of our manuscript and for their constructive and suggestive comments, which have helped improve the overall quality of our manuscript. Point-by-point responses to all comments by the academic editor and reviewer's have been prepared and provided below.

We hope that these revisions are sufficient to make our manuscript suitable for publication in PLOS ONE and look forward to hearing from you at your earliest convenience.

Sincerely,

Toshinobu Kawai

Institute of Health and Sport Sciences, University of Tsukuba

1-1-1 Tennodai Tsukuba Ibaraki 3058574 Japan

Telephone: +81-80-5699-9789

Fax: +81-29-853-6507

E-mail: kawai.toshinobu.gp@u.tsukuba.ac.jp

**Response to Academic Editor**

**Academic Editor (1):** *Ensure that your manuscript meets PLOS ONE's style requirements.*

**Response (1):** We have carefully checked PLOS ONE's style requirements and modified the typeface of Heading 3 to match the template. We checked the file name carefully prior to uploading the file.

**Academic Editor (2):** *We notice that your supplementary figures are uploaded with the file type 'Figure'. Please amend the file type to 'Supporting Information'.*

**Response (2):** In accordance with the journal requirements, the file name of the supplementary figure was changed to 'supporting information' and uploaded.

**Response to Reviewer #1**

Thank you for taking the time to review this manuscript. The reviewer's comment was, "Overall, the manuscript appears to be well-prepared and meets the standards for publication." Based on this comment, no particular points needed to be addressed.

The evaluation of Data Availability was "No", but we understand that this is due to the fact that "No-some restrictions will apply" was stated in our Data Availability section. The data for the current study is not publicly available, but can be provided upon reasonable request and with the approval of the relevant department in Koshu City.

**Response to Reviewer #2**

Thank you for evaluating each component of this manuscript. Overall, the comments were positive, and no particular points were needed to be addressed.

**Response to Reviewer #3**

We appreciate your detailed comments and advice to improve the overall quality of this manuscript. We believe that the unclear descriptions are now improved. We have provided relevant explanations for all incorporated revisions.

**Introduction (1):** *The introduction provides a comprehensive background on depression in adolescents. In line 37, the included study with 3331 children was conducted in 2004, and the other research (line 41) was conducted in 2016. It would be better to connect this information as a trend. This will enable the reader to comprehend the trend of depression over the years.*

**Response - Intro. (1):** We agree that the different time points at which the two articles were reported are crucial information. In line 38, we stated that the study in [2] was conducted in 2004 and that the DSRS-C scores of Japanese children are consistent in lines 42-43.

**Introduction (2):** *The percentage of depression in the DSR-C studies was mentioned. In line 43, the percentages of depression were more significant in Europe and the USA. However, providing more specific information by including figures would be helpful. This would contextualise the prevalence of depression locally and worldwide.*

**Response - Intro (2):** We agree that stating the relevant values will enhance the reader's understanding. Accordingly, we have stated the report of Ivarsson et al. (1994) in lines 44-46 as an example in the revised version of the manuscript.

**Introduction (3):** *The symptoms of depression were mentioned in the second paragraph - however, the sentence in line 50 needs paraphrasing to deliver the intended point.*

**Response - Intro (3):** We agree that the clarity of the original statement was poor and warranted clarification. Accordingly, this statement was rephrased to improve the clarity and readability (lines 51-54). Thank you for highlighting this issue.

**Introduction (4):** *In this third paragraph, the recommended duration of physical activity is documented. However, it would also be better to mention the recommended exercise type. (frequency and intensity). This will provide a whole picture of the physical activity recommendation.*

**Response - Intro (4):** The wording to indicate the recommended types and frequency of exercise was hard to understand and has been corrected in lines 62-70. Note that the Japanese guideline introduces a diverse range of physical activity types and, therefore, does not provide recommended conditions for specific exercise intensity. This point was reinforced by the WHO recommendation.

**Introduction (5):** *In the last paragraph, bukatsu sport was mentioned. It would be helpful to briefly describe this sport and say if it is optional or mandatory for the students. This will keep the reader on track.*

**Response - Intro (5):** Thank you for highlighting this issue. We agree that for those who do not live in Japan, imagining the specifics of *bukatsu* could be difficult, warranting the addition of further information. In lines 87-88, we emphasize that although *bukatsu* is not mandatory, many students participate in it. Additionally, we have listed the names of specific sports in *bukatsu* in line 91.

**Methods (1):** *This study includes massive and structured data, making the results more reliable. However, it was initially challenging to comprehend whether the study was retrospective or both retrospective and prospective. For example, In the 3rd paragraph, the author explains that a self-administered questionnaire was given to the participants. Was this done by the Koshu project or from your side by interviewing the subjects? Please clarify these points to be easier for the reader to follow.*

**Response - Methods (1):** This study was a prospective assessment, obtaining and analyzing cohort data conducted by the Koshu Project. The wording in the third paragraph was unclear; hence, the content was revised in lines 133-137.

**Methods (2):** *In the participants and procedure, the data were collected from the Koshu project. Out of these, 1890 were selected. Does this represent all the available data, or was a sample size calculated? Please specify this information to comprehend the methodology.*

**Response - Methods (2):** The 1,890 represents the number of all available data. We have modified the relevant statement (line 124) to indicate that this value indicates the overall data.

**Methods (3):** *Under the section depressive symptoms, some of the studies used 15 as a cut-off point. Please provide a more substantial justification if possible. This might underestimate the depressive symptoms, and it would be difficult to compare it with other international studies.*

**Response - Methods (3):** Even if the same scale is employed, different evaluation criteria will warrant careful discussion of the results; however, our manuscript did not consider this point. The rationale for setting the cutoff point at 16 is clarified in lines 148-153.

**Methods (4):** *Does the physical activity time mean the duration or the timing? In the methods, the question refers to the duration; however, the table refers to the timing, as there is another question for the activity based on the duration spent in the physical activity. The world time is, therefore, overlapping between the timing and the duration.*

**Response - Methods (4):** Physical activity time represents the duration for which physical activity was performed during the week. In Table 1, [hours:minutes] was used to represent the duration of physical activity time, but since the question asked " How many hours a week do you usually spend on physical activities... " and the decimal point was used in the text as well, the minutes were unified to be represented by a decimal point.

**Methods (5):** *Figure 1 shows the recruitment done in this study, which is a great visual explanation. However, it must be self-explanatory to make it easier for the reader to follow.*

**Response - Methods (4):** The wording in the top box of Fig 1 was modified to clearly indicate that the data represent ALL available participants.

**Methods (6):** *Table 1 divides body image into wanting to be fat, wanting to stay the same and wanting to be thin. However, the text line 172 mentioned that it would be classified into two categories: want to be thin and don't want to be thin. Please change the results in the table to be consistent.*

**Response - Methods (6):** As pointed out, "want to be fat" and "want to stay the same" in Table 1 were merged and classified into two categories: "want to be thin" and "do not want to be thin."

**Methods (7):** *Table 1, including physical activity time. Please specify if it is referred to as timing or duration, as the numbers indicate timing, whereas the explanation in the text relates to duration. Please justify.*

**Response - Methods (7):** Point 4 above clarifies the implication of physical activity time. The wording in Table 1 has been modified from [hours:minutes] to a notation of time, including a decimal point.

**Discussion (1):** *It is better to interpret the discussion in one context.*

**Response - Discussion (1):** To comprehensively understand the results of this study, we thought it would be appropriate to discuss the results of the sub-analysis before discussing the main analysis results; hence, we structured the argument in this manner. A relevant explanation has been provided in lines 336-338.

**Discussion (2):** *In the participant characteristics, why is the DSRS-c lower than in the previous studies? Given the difference in time and era, you need to justify the possible causes.*

**Response – Discussion (2):** It is difficult to explain structurally why the rate of depressive tendencies among the participants in this study is lower than in other previous studies in Japan based on the data of this study alone. Nevertheless, in lines 308-313, we discuss the potential of the social environment in Koshu City and the duration of physical activity, which is discussed later. Regarding the difference in time periods noted by reviewer 3, we explained in the Introduction (lines 42-43) that this is consistent in Japan.

**Discussion (3):** *It is helpful to include studies that examine the depressive symptoms from childhood to early adulthood to exclude confounding factors*

**Response - Discussion (3):** While some studies have found that age factors are not associated with depressive symptoms in adolescents [38], a 2018 Japanese study [32] identified significant differences by age, which may be a confounding factor. The current study did not assess this effect itself because it excluded data from children with depressive tendencies at age 11. This has been explained in the Limitations of the Study section (lines 425-430). Relevant references were added to the list of references [38].

**Conclusion:** *The conclusion is comprehensive. In line 455, further studies are needed before concluding that poor social interactions cause depressive symptoms and decreased physical activity time. It is favourable to add a recommendation in this regard.*

**Response - Conclusion:** As pointed out, further research is needed to comprehensively elucidate these relationships, and we have stated the need for such research in lines 453-456.

**Some additional modification:** Some spelling errors and inappropriate wording were found and corrected.
