# Supplementary material for: Association between physical activity duration and depressive symptoms in adolescents: A longitudinal study in a rural city in Japan
Source: PLoS One. 2024 May 31;19(5):e0304783. doi: 10.1371/journal.pone.0304783 (PMC11142661; doi:10.1371/journal.pone.0304783)
Supplement: Attachment — Submitted filename: Response to Reviewers #2.docx [file pone.0304783.s003.docx]

May 14, 2024

Dr. Mukhtiar Baig

Editor

PLos One

Dear Dr. Baig,

Re: Resubmission of manuscript No. PONE-D-23-34588R1

We wish to re (2) -submit the manuscript titled "Association between physical activity duration and depressive symptoms in adolescents: A longitudinal study in a rural city in Japan." (We have slightly changed the title)

We sincerely thank you and the reviewers for their careful assessment of our manuscript and for their constructive and suggestive comments, which have helped improve the overall quality of our manuscript. Point-by-point responses to all comments by the reviewer's have been prepared and provided below.

We hope that these revisions are sufficient to make our manuscript suitable for publication in PLOS ONE and look forward to hearing from you at your earliest convenience.

Sincerely,

Toshinobu Kawai

Institute of Health and Sport Sciences, University of Tsukuba

1-1-1 Tennodai Tsukuba Ibaraki 3058574 Japan

Telephone: +81-80-5699-9789

Fax: +81-29-853-6507

E-mail: kawai.toshinobu.gp@u.tsukuba.ac.jp

**Response to Reviewer #3**

We appreciate your detailed comments and advice. We believe that your suggestion improves the overall quality of this manuscript.

**Abstract:** *In line 22, physical activity was abbreviated as PA; however, in line 24, the full sentence was used. Please choose whether to consistently use the abbreviation or the full term. This will ensure writing consistency and provide clarity of purpose for the abbreviation.*

**Response - Abstract:** Unified to full sentence (physical activity).

**Introduction (1):** *The term "physical activity" is written in full in line 57, 75, 76, and elsewhere. Please use the search function to replace the full term with "PA" if you decide to keep the abbreviation.*

**Response - Introduction (1):** In the whole manuscript, "physical activity" was used as a complete term. In the figures and tables, the full term was also used in basic. Abbreviations (PA) were used due to space limitations; explanations were provided outside the tables and in the captions.

**Introduction (2):** *In line 70, please leave a space between "5-17" and "years old."*

**Response - Introduction (2):** Space was inserted in line 68.

**Introduction (3):** *It is recommended to define "Bukatsu" immediately after its introduction in line 68 rather than later in line 72 to maintain reader comprehension.*

**Response - Introduction (3):** Moved the description part of the *bukatsu* to one-sentence before (lines 88-90).

**Methods (1):** *Please mention ethical considerations either at the beginning or end of the methods section.*

**Response - Methods (1):** Ethical considerations were described in the Participants and Procedure section, including the approval number of the ethical review (lines 144-146).

**Methods (2):** *Clarify whether both retrospective (lines 103-139) and prospective approaches (lines 143-150) were used.*

**Response - Methods (2):** Described a retrospective analysis to examine physical activity patterns and depressive symptoms from 11 y to 14 y, and a prospective analysis to observe progression of depressive symptoms and physical activity patterns in lines 131-135.

**Methods (3): *S****pecify the number of schools visited and the total sample size from these schools. If the sample size is included from the1890, please indicate this.*

**Response - Methods (3):** All public elementary and junior high schools (13 elementary schools and 5 junior high schools) in Koshu City are participating in the project. The number of each school is included in line 130. The project was conducted by the Koshu City Board of Education, and the researchers themselves did not visit the schools during the survey. The flow diagram of the sample used for the analysis from the 1890 responses is shown in Fig. 1.

**Methods (4):** *Physical Duration - In some contexts, "time" and "duration" can be used interchangeably, but they don't always mean the same thing and might cause confusion. "Time" generally refers to a specific point, while "duration" specifically refers to the length of time that something lasts. Therefore, the word "duration" would be more suitable in the context of your research.*

**Response - Methods (4):** Unified to Physical activity "duration" instead of physical activity "time." The title of this manuscript was also changed from "time" to "duration."

**Results (1):** *Please specify physical activity duration in the table as hours and minutes.*

**Response - Results (1):** The notation was reverted to hrs:min and the units are given in the table.

**Results (2):** *Consider changing the word "time" to "duration" to eliminate confusion for the reader.*

**Response - Results (2):** The terminology was changed from "time" to "duration" in all parts of this manuscript.
